# Microglia-dependent neuroprotective effects of 4-octyl itaconate against rotenone-and MPP+-induced neurotoxicity in Parkinson’s disease

**DOI:** 10.1038/s41598-023-42813-8

**Published:** 2023-09-20

**Authors:** Ning Xia, Victoria Madore, Ali Albalakhi, Sonia Lin, Taylor Stimpson, Yuehang Xu, Michael A. Schwarzschild, Rachit Bakshi

**Affiliations:** 1 Molecular Neurobiology Laboratory, Massachusetts General Hospital, Boston, MA 02129 USA; 2 Harvard Medical School, Boston, MA 02115 USA

**Keywords:** Cellular neuroscience, Cell biology, Neuroscience

## Abstract

Chronic neuroinflammation is implicated in the pathogenesis of Parkinson’s disease (PD), one of the most common neurodegenerative diseases. Itaconate, an endogenous metabolite derived from the tricarboxylic acid cycle via immune‐responsive gene 1 activity, may mediate anti-inflammatory responses by activation of the nuclear factor erythroid 2-related factor 2 (Nrf2) antioxidant pathway. This study investigates the neuroprotective potential of 4-octyl itaconate (OI), a cell-permeable derivative of itaconate, in cellular models of PD. OI not only suppressed lipopolysaccharide-induced proinflammatory cascades of inducible nitric oxide synthase, cyclooxygenase-2, and cytokines release in mouse BV2 microglial cells but also activated the Nrf2 signaling pathway and its downstream targets in these cells. Conditioned medium derived from OI-treated BV2 cells protected against rotenone- and MPP^+^-induced neurotoxicity in Neuro 2A cells. Overall, our findings support the anti-inflammatory neuroprotective potential of OI in PD.

## Introduction

Parkinson’s disease (PD) is a progressive neurodegenerative disorder leading to the death of dopaminergic (DA) neurons in the substantia nigra pars compacta (SNpc) resulting from a combination of genetic and environmental factors^1,2^. Although the critical molecular and cellular events underlying DA cell death are unclear, inflammation may contribute over time. Microglia activation and peripheral immune cell infiltration may contribute to the induction and aggravation of neuroinflammation in PD, with substantia nigra reported to have the highest density of microglia among different brain regions with more than twice that compared to cortex^3^. Moreover, midbrain DA neurons exhibit more sensitivity to the death-inducing properties of cytokines such as tumor necrosis factor (TNF) than neurons in the hippocampus or cortex^4,5^. Therefore, targeting the inflammatory processes is a promising intervention strategy for drug development to help ameliorate PD progression.

Itaconate, an endogenous metabolite generated by the tricarboxylic acid (TCA) cycle, is derived from cis-aconitate decarboxylation that is catalyzed by immune-responsive gene 1 (IRG1) in the mitochondrial matrix. The anti-inflammatory and anti-immune mechanisms of itaconate have been described recently^6,7^. Previous studies have demonstrated that itaconate is generated in response to (lipopolysaccharide) LPS in macrophages and promotes an anti-inflammatory response by activating the nuclear factor erythroid 2-related factor 2 (Nrf2) pathway. 4-Octyl itaconate (OI), a cell-permeable itaconate derivative, has been shown to exert anti-inflammatory effects by targeting glyceraldehyde 3-phosphate dehydrogenase to decrease aerobic glycolysis in macrophages^8^. Intraperitoneal administration of dimethyl itaconate (DMI), another itaconate derivative, upregulates heme oxygenase-1 (HO-1) expression levels in microglia and ameliorates brain injury in ischemic stroke models^9^. The addition of itaconate to reperfusion fluids after mouse cerebral ischemia/reperfusion injury increased glutathione levels and reduced reactive oxygen/ nitrogen species (ROS/RNS) to improve neurological function^10^. These findings suggest therapeutic opportunities to use itaconate or its derivatives to target neuroinflammation in PD. Hence, in this study, we aimed to investigate the immunomodulatory effects of OI and elucidate the molecular mechanisms underlying the protective effect of OI in a cellular model of PD.

## Results

### OI suppresses the pro-inflammatory response to LPS in BV2 cells

**Figure 1:**
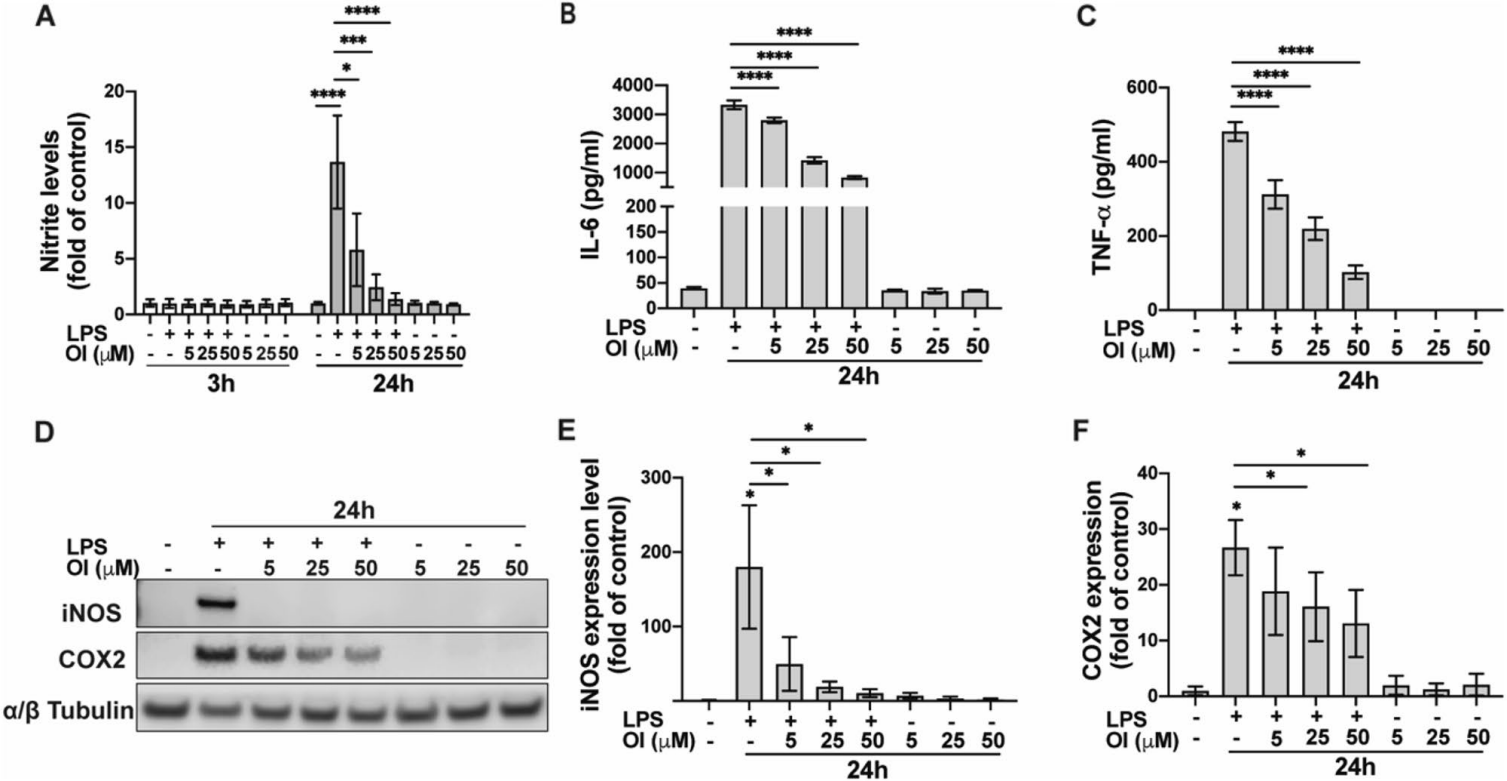
Itaconate suppresses inflammatory reactions in activated microglia. (**A**–**F**) BV2 cells were exposed to LPS ± OI for 3 or 24 h at indicated concentrations. (**A**) Nitrite level in supernatant 3 h and 24 h after indicated treatment was tested by Griess assay (mean ± SEM, one-way ANOVA, *p < 0.05, ***p < 0.001, ****p < 0.0001; n ≥ 3 independent experiments). (**B**,**C**) Supernatant of BV2 cells was collected 24 h after treatment to detect the release of different inflammatory cytokines, including IL-6 and TNF-α in the medium by ELISA assay (mean ± SEM, one-way ANOVA, ****p < 0.0001; n = 4 independent experiments). (**D**) Representative Western blot images of COX2 and iNOS protein levels 24 h after treatment as indicated from three independent experiments. α/β tubulin was used as a loading control. Bar graph representing the quantification of iNOS (**E**) and COX2 (**F**) normalized to α/β tubulin (mean ± SEM, one-way ANOVA, *p < 0.05; n = 3 independent experiments).

We first validated the effect of OI treatment on the suppression of proinflammatory response in microglial cells. We performed a dose-response curve to test the nitrite release from BV-2 cells at 24 h by Griess assay by using several doses of LPS. Our data showed that LPS at 100 ng/ml can significantly increase nitrite release without significant cell death (Fig. S1). 100 ng/ml LPS was used to activate BV2 cells to mimic the overactivated microglia in our in vitro experiments. We examined the nitrite concentration as a correlate of microglial activation in the culture medium of BV2 cells. A dose-response curve of OI showed no changes in BV2 cell viability by MTS assay at 24 h (Fig. S2). No significant changes in nitrite levels were observed 3 h after treatment of LPS with or without OI. However, after 24 h, a highly significant increase in nitrites production was induced by LPS, which was dramatically limited by the cotreatment with OI in a dose-dependent manner (Fig. 1A). IL-6 and TNF-α levels in the supernatant were significantly elevated 24 h after LPS treatment. OI treatment suppressed the levels of these cytokines in a dose-dependent manner (Fig. 1B–D). Inducible nitric oxide synthase (iNOS) and cyclooxygenase-2 (COX2) are classical pro-inflammatory markers. To further validate the anti-inflammatory effect of OI, protein levels of iNOS and COX2 were quantified by western blot. At an early time point (3 h after treatment), only COX2 was upregulated by LPS. Surprisingly, OI showed no effects on COX2 protein level at the 3 h time point.

Meanwhile, iNOS was not detectable at 3 h (data not shown). However, 24 h after treatment, LPS-dependent elevation of iNOS and COX2 proteins were significantly suppressed by OI in a dose-dependent manner (Fig. 1D–F). All these data strongly support the anti-inflammatory properties of OI in LPS-activated microglia cells.

### OI boosts Nrf2 activation in the LPS-activated BV2 cellular model

**Figure 2:**
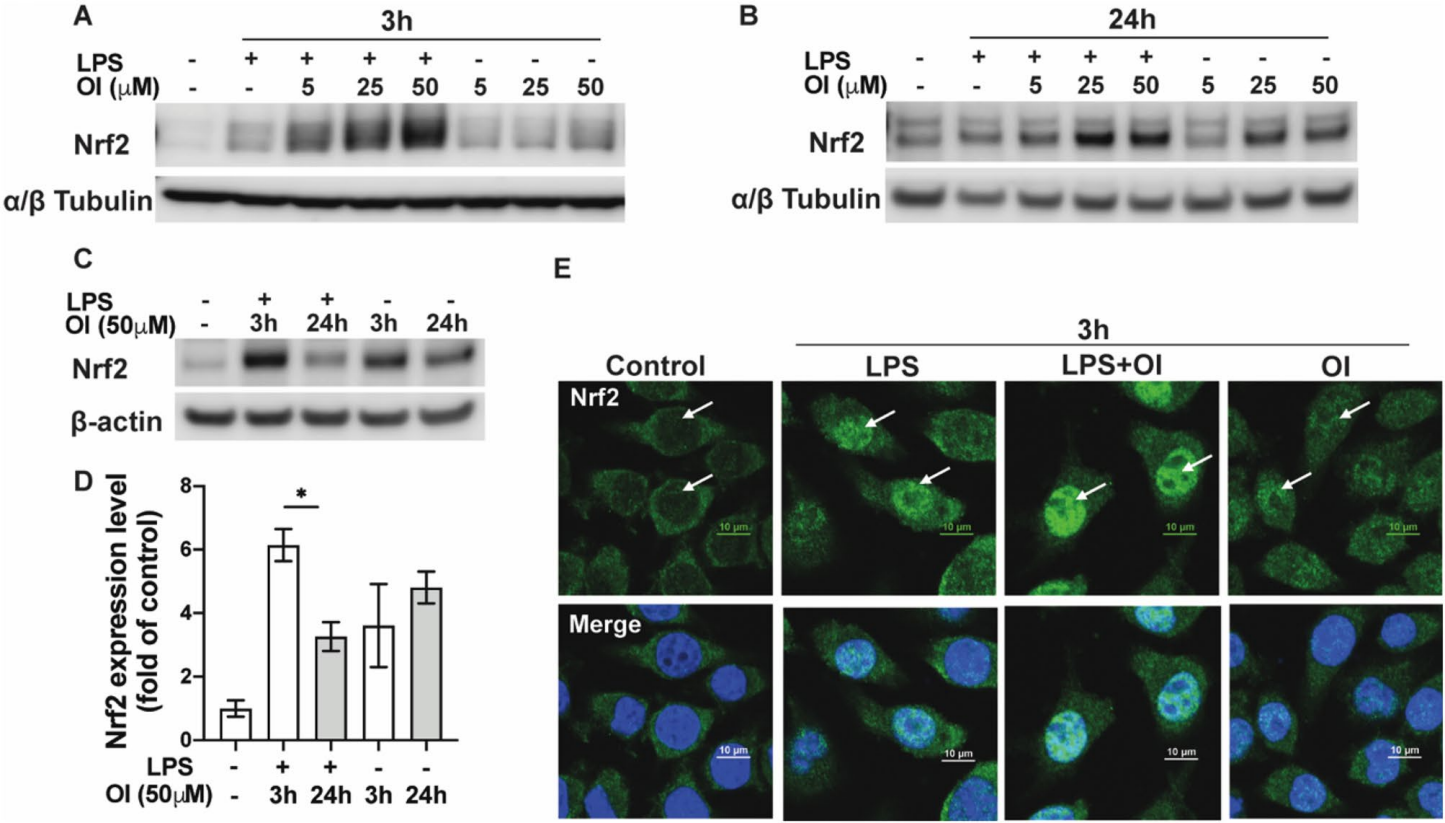
Activation of Nrf2 pathway by OI in activated microglia. BV2 cells were exposed to different concentrations (5, 25, 50 μM) of OI ± 100 ng/ml LPS for 3 h and 24 h. Nrf2 protein levels in the whole cell were tested by western blot at 3 h (**A**) and 24 h (**B**). The blots shown are representative images from at least three independent experiments. α/β tubulin was used as a loading control. (**C**) Western blot analysis compared Nrf2 expression after being exposed to LPS with 50 μM OI at 3 h and 24 h. β-actin was used as a loading control. Western blot images are representative of three independent experiments. (**D**) The bar graph represents the quantification of Nrf2 expression normalized to β-actin in (**C**) (mean ± SEM, one-way ANOVA, *p < 0.05; n = 3 independent experiments). (**E**) Representative confocal images of Immunofluorescence staining of Nrf2 (green) 3 h after treatment. The arrow shows the induction and translocation of the Nrf2 protein in cells.

It has been reported that itaconate activates Nrf2 by alkylation of a KEAP1 cysteine residue in microphages^6^. To confirm these findings in our microglial model, we analyzed Nrf2 protein levels by western blot at both 3 h and 24 h time points (Fig. 2A and B). LPS treatment as an inflammatory stressor caused a slight increase in Nrf2 levels in BV2 cells. However, co-treatment with OI significantly upregulated the Nrf2 protein expression in a dose-dependent manner compared to treatment with LPS or OI alone. Nrf2 protein levels were much higher at 3 h than at 24 h, indicating Nrf2 activation as an early event during OI treatment (Fig. 2C and D). We also monitored the nuclear translocation of Nrf2 by immunofluorescence confocal microscopy. We observed a significant redistribution of Nrf2 immunoreactivity to a predominantly nuclear localization in the OI+ LPS group compared to the LPS group at 3 h (Fig. 2E). These data suggest that OI significantly upregulates Nrf2 levels in microglial cells.

### OI regulates Nrf2 target genes

**Figure 3:**
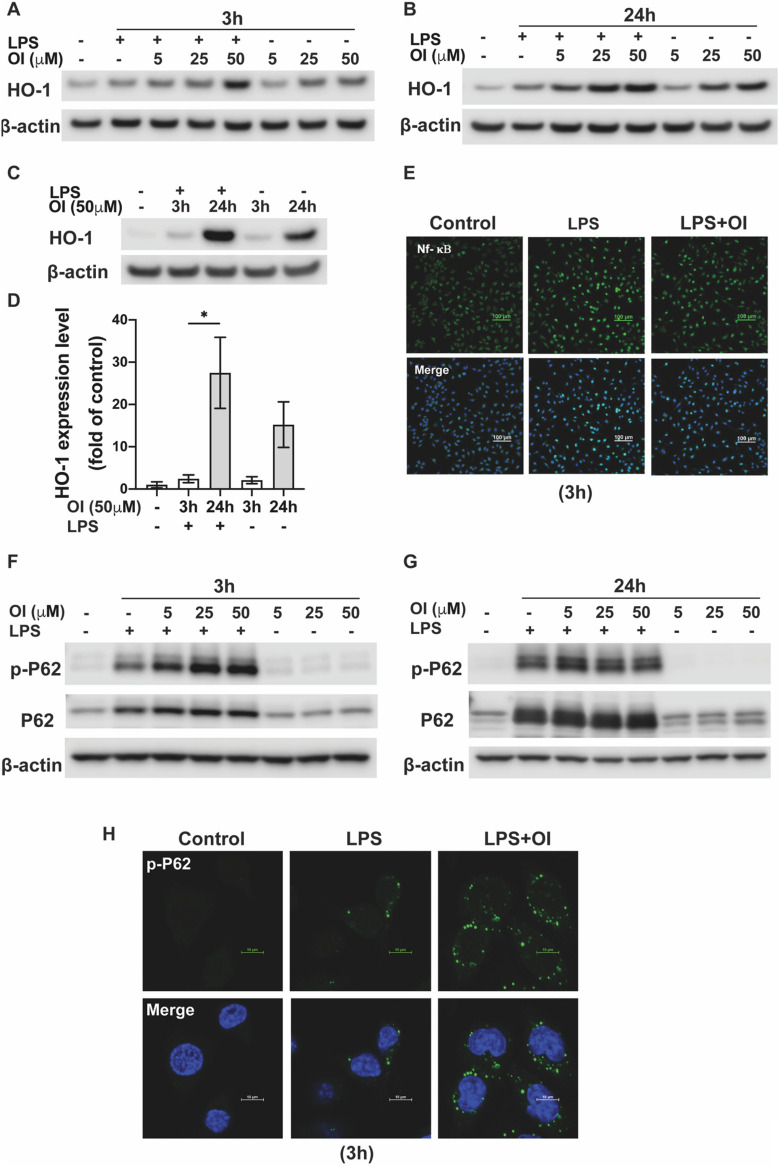
Regulation of HO-1/P62/Nf-κB pathway by OI in activated microglia. BV2 cells were exposed to different concentrations (5, 25, 50 μM) of OI ± 100 ng/ml LPS for 3 h and 24 h. HO-1 protein levels in the whole cell were tested by western blot at 3 h (**A**) and 24 h (**B**). β-actin was used as a loading control. (**C**) Western blot analysis compared HO-1 expression after being exposed to LPS with 50 μM OI at 3 h and 24 h. β actin was used as a loading control. (**D**) The bar graph represents the quantification of HO-1 expression normalized to β-actin in (**C**) (mean ± SEM, one-way ANOVA, *p < 0.05; n = 3 independent experiments. (**E**) Representative confocal images of Nf-κB fluorescence staining 24 h after treatment. Western blot analysis of phospho-P62 and total P62 protein levels 3 h (**F**) and 24 h (**G**) after treatment indicated. β-actin was used as a loading control. (**H**) Representative confocal images of p-P62 fluorescence staining 3 h after treatment.

To investigate the underlying mechanism of Nrf2 activation by OI, we further evaluated upstream and downstream molecular pathways of Nrf2. HO-1, an antioxidant enzyme primarily regulated by Nrf2, was increased in LPS and OI groups, consistent with an increase in Nrf2 levels (Fig. 3A and B). Significantly, OI activated Nrf2 at the early time point (3 h), leading to an increase in downstream target HO-1 at the later time point (24 h) (Fig. 3C and D). NF-κB dysregulation has been shown to be related to neuroinflammation in the brain of Parkinsonian patients^11,12^. Microglial activation of NF-κB plays a central role in the release of reactive oxygen species and proinflammatory cytokines (such as IL-1β, interferon-γ, and TNF-α) that can cause secondary neurotoxicity^4,13^. We found that Nf-κB was significantly induced and translocated into the nucleus by LPS both 3 h and 24 h after treatment. OI inhibited NF-κB nuclear translocation at 24 h, suggesting a possible modulation by earlier activation of the Nrf2/HO-1 pathway (Fig. 3E). P62 has been shown to sequester Keap1 to autophagic degradation, ultimately leading to the stabilization of Nrf2 and the transactivation of Nrf2-dependent genes^14^. The phosphorylated and total protein levels of P62 were markedly elevated higher by OI in the LPS induced BV2 cells at both 3 h and 24 h compared to the LPS group, indicating a possible autophagy mechanism is involved (Fig. 3F and G). The result was further validated by fluorescence staining (Fig. 3H).

### OI protects neurons from PD toxins by altering microglia response

**Figure 4:**
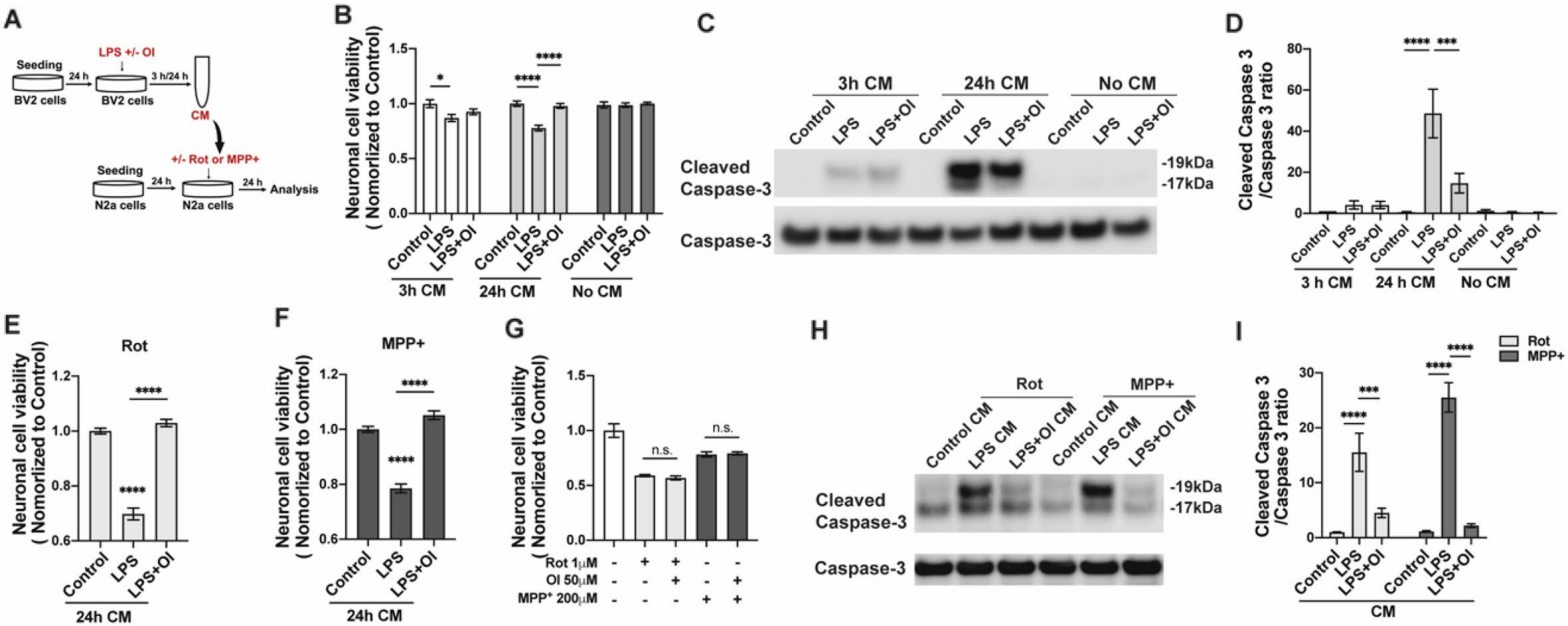
Conditioned medium from OI-treated BV2 cells protects DA neurons from toxin-induced cell death. Conditioned medium (CM) from control/LPS/LPS+ 50 μM OI treated BV2 cells were collected after 3 h, and 24 h. All neuronal cell viability was tested by MTS assay (**A**) Schematic of the CM collection and treatment. (**B**) N2a cells were treated as indicated for 24 h (mean ± SEM, one-way ANOVA, *p < 0.05, ****p < 0.0001; n > 5 independent experiments). (**C**) N2a cells were treated with 3 h CM, 24 h CM, or LPS ± OI for 24 h. Western blot analyzed cleaved Caspase -3 and Caspase-3 expressions in whole-cell lysis. The representative image was from 3 independent experiments. (**D**) The bar graph represents the quantification of the Cleaved Caspase -3/Caspase -3 ratio (mean ± SEM, one-way ANOVA, ***p < 0.001, ****p < 0.0001; n = 3 independent experiments). (**E**) N2a cells were treated with 1 μM rotenone with indicated 24 h CM (mean ± SEM, one-way ANOVA, ****p < 0.0001; n = 5 independent experiments). (**F**) N2a cells were treated with 200 μM MPP^+^ with indicated 24 h CM (mean ± SEM, one-way ANOVA, ****p < 0.0001; n = 5 independent experiments). (**G**) N2a cells were treated with rotenone with/without OI or MPP+ with/ without OI for 24 h (mean ± SEM, one-way ANOVA, n = 5 independent experiments). (**H**) N2a cells were treated with MPP+ or rotenone with/ without the 24 h CM as indicated for 24 h. Western blot analyzed cleaved Caspase -3 and Caspase-3 expressions in whole-cell lysis. The representative image was from more than 3 independent experiments. (**I**) Bar graph representing the quantification of Cleaved Caspase -3/Caspase -3 ratio (mean ± SEM, one-way ANOVA, ***p < 0.001, ****p < 0.0001; n = 3 independent experiments).

Neuro 2A (N2a) cells, a mouse neural crest-derived cell line that has been extensively used to study PD, was used as a cellular model in this study^15^. To test the impact of the anti-inflammatory effect of itaconate on neuronal cells, we collected different conditioned medium (CM) from BV2 cells treated with LPS, with or without OI, for 3 h and 24 h, respectively. N2a cells were treated with CM from all groups for 24 h (Fig. 4A). LPS CM was toxic to neurons at both 3 h and 24 h collected media. LPS+ OI CM shows a significant rescue in 24 h CM (Fig. 4B). As a control, direct treatment of N2a cells with LPS or OI or a combination of both does not affect neuronal viability (Fig. 4B). Caspase-3 has been identified as a key mediator of apoptosis in neuronal cells. Western blot results show that both 3 h LPS CM and 24 h LPS CM increased cleaved caspase-3 levels. The increased cleaved caspase-3 protein levels were dramatically reduced in the 24 h-LPS+ OI CM group (Fig. 4C and D). We further applied two DA neuron toxins (rotenone and MPP+) to N2a neuronal cells. Based on the dose-dependent toxicity of rotenone and MPP+ on N2a cells after 24 h (data not shown), 1 μM rotenone and 200 μM MPP+ were used in the experiments. LPS+ OI CM showed significant neuronal protection against rotenone or MPP+ toxicity as indicated by higher cell viability compared to LPS CM (Fig. 4E and F). However, the direct treatment of OI on N2a cells did not impact rotenone or MPP+ toxicity (Fig. 4G). Consistent with the cell survival results, under both MPP+ and rotenone exposure, the 24 h LPS+ OI CM shows a remarkably decreased cleaved caspase-3 compared to the 24 h-LPS CM group (Fig. 4H and I). Our data suggest a microglia-dependent protective effect of itaconate.

## Discussion

In PD, neither the cause nor the mechanisms underlying the death of the nigrostriatal DA neurons are known. Since neuroinflammation may contribute to the degeneration of these neurons, targeting neuroinflammation could help prevent PD or slow its progression. In recent years researchers have found that itaconate, a metabolite of the TCA cycle, exhibits crucial anti-inflammatory effects via Nrf2 activation in macrophages. The present study confirmed that OI, one of the itaconate derivatives, suppressed nitrite production and cytokines release in LPS-treated microglial cells in vitro. Consistent with the previous studies in macrophages, we found OI boosted Nrf2/HO-1 protein expression induced by LPS in microglia. Our data also revealed that the CM from microglia treated with LPS was toxic to neurons and that this toxicity was eliminated if the CM from microglia had also been treated with OI. Using different PD toxins, the CM from microglia treated with OI showed similarly significant protective capacity by attenuating neuronal loss. Taken together, our in vitro data convincingly support the microglia-dependent neuroprotective role of OI.

Recently itaconate’s role as a novel physiological inflammation regulatory metabolite has been demonstrated in research studies. Lampropoulou et al. first described the anti-inflammatory effect of itaconate in vitro and in vivo during macrophage activation through inhibition of succinate dehydrogenase^16^. After that, multiple studies have reported the therapeutic anti-inflammatory effect of itaconate in a variety of disease models. In mouse models of sepsis, OI has been shown to prolong survival and decrease IL-1β and TNF release in response to LPS challenge^6,8^. OI reduced IL-1β release induced by LPS in peripheral blood mononuclear cells (PBMCs) isolated from cryopyrin-associated periodic syndrome patients^17^. So far, although studies about the therapeutic potential of itaconate in peripherical immune responses have been extensively studied, few have been done in the central nervous system. Kuo et al. reported that DMI suppresses microglial activation and prevents the CNS infiltration of Th1 and Th17 cells in experimental autoimmune encephalomyelitis^18^. The induction of IRG1 in microglia following ischemic stroke serves as an endogenous protective mechanism to restrain brain injury in rodent models^9^. Several cell-permeable derivatives of itaconate, such as DMI, OI, and ethyl itaconate (4-EI), have been synthesized to imitate the action characteristics of endogenous Itaconate. OI is considered a better derivative for itaconate because, like endogenous itaconate, it has lower thiol reactivity making it more suitable to probe the physiological function of itaconate^6,19^. DI and OI have been implicated in similar microglia suppression in chronic pain and spinal cord injury mouse models, respectively^20,21^.

Neuroinflammation has been implicated in PD pathophysiology, with increased proinflammatory cytokines levels observed in the blood, cerebrospinal fluid (CSF), and the brain postmortem of PD patients. Microglia are the innate immune cells of the central nervous system, which are considered the first line of defense against invading pathogens. In our study, we used LPS-stimulated BV2 microglial cells as a cellular model to mimic the activated microglia pathology in PD. We observed robust anti-inflammatory effects from OI against LPS challenge in microglia cells in agreement with previous findings. Studies indicate that NO can influence the regulation of the TCA cycle and the accumulation of itaconate in macrophages^22,23^. The interplay between itaconate and NO may contribute to the regulation of inflammation in our study. It is likely that the increased NO disturbed the TCA cycle metabolic homeostasis, which is restored by the exogenous OI.

Nrf2 is a transcription factor that plays a crucial role in cellular defense against oxidative stress and neuroinflammation in CNS. In MPTP mouse models, Nrf2 modulates microglial dynamics and control’s microglial function^24^. These findings suggest much interest in Nrf2 as a promising target against neuroinflammation in PD. Multiple pathways of itaconate have been identified at present. Alkylation of Keap1 to activate Nrf2 has been widely studied and emphasizes the importance of Nrf2 during itaconate function. Both DI and OI have been found to activate Nrf2 in macrophages^6,7^. Our results demonstrated that OI activated Nrf2 by increasing its expression levels in microglia. More importantly, OI boosted total Nrf2 protein levels in the presence of LPS treatment, indicating the role of Nrf2 during the protection process of OI against inflammation. In this study, we observed that LPS induced Nrf2 translocation into the nucleus in microglia, which is a general adaptive mechanism when cells are provoked by stress. Unfortunately, this activation of the Nrf2 pathway is not sufficient to protect neuronal cells via the non-cell autonomous pathway. We demonstrated that HO-1 expression levels were boosted by OI, like Nrf2. By examining two different time points, we found OI increased the expression level of Nrf2 earlier than HO-1, further implicating the role of HO-1 as downstream of Nrf2 activated by OI in microglia. NF-κB is widely expressed in the central nervous system and is a crucial regulator of inflammatory and immune responses and the expression of multiple genes such as iNOS and COX2. It also promotes the secretion of various proinflammatory cytokines, such as TNF-α and IL-1β^25^. Under physiological conditions, NF-κB binds to its inhibitor I-κB and stays dormant in the cytoplasm. Once activated, it dissociates from I-κB and translocates to the nucleus, where it initiates the transcription of downstream genes. Our data shows that OI significantly reduced NF-κB migration into the nucleus, supporting NF-κB as a downstream target of OI. P62 is a receptor for ubiquitinated substrates sequestered into autophagosomes, and it regulates protein aggregate formation^26^. Indeed, P62 is the major component of the ubiquitin-containing inclusions in various neurodegenerative diseases such as PD^27,28^. The adaptor protein P62 regulates Nrf2 levels. Phosphorylated P62 displaces Nrf2 and binds KEAP1, causing the accumulation of Nrf2. On the other hand, Nrf2 also induces the expression of P62 through transcriptional activation^29^. In microglial cells, we observed an intense elevation of both phosphor-P62 and total P62 by OI in the presence of LPS. Our new findings suggest that a P62/Nrf2/HO-1/NF-κB axis is a pathway involved in the anti-inflammatory function of OI in microglia, supporting the possible regulation of itaconate as a natural metabolite protectant against PD.

Emerging evidence shows that microglial dysfunctions contribute to PD pathogenesis and progression by both the loss of normal homeostatic functions and the gain of neurotoxic functions^30–33^. Activation of Nrf2 has been confirmed to provide non-cell autonomous protection to nearby cells^34,35^. To study the microglia-neuron interactions after OI treatment, we applied a simplified co-culture setting published before^36,37^ by growing N2a neuronal cells in BV2 microglial conditioned medium. We didn’t observe any direct toxicity of LPS on N2a cells or BV2 cells based on the MTS assay. As shown in our data, the supernatant from the LPS-activated BV2 cells contains different proinflammatory cytokines including TNF-α and IL-6, both of which have been reported can cause neuron death. In vitro study, IL-6 was reported to cause neuronal cell death^38^. And in vivo, the upregulation of IL-6 exacerbates dopaminergic degeneration in the 6-hydroxydopamine- (6-OHDA) induced PD rats model^39^. Overexpression of dominant-negative TNF-α specifically inhibits TNF signaling in the SNpc and attenuates activation of microglia, thereby halting the progressive loss of nigral dopaminergic neurons and attenuating behavioral deficits in 6-OHDA-induced rat PD model^40,41^. Here, we have found that the CM from LPS-activated BV2 microglial cells induces N2a neuronal death. And the CM from the OI-treated BV2 cells shows significantly higher neuronal cell viability and lower cleaved caspase-3 protein expression, indicating a neuroprotective benefit. We also exposed the rotenone or MPP+ treated N2a neurons with the CM will mimic a microenvironment for neurons with cytokines or factors released by microglia and confirmed the neuroprotective effect of the OI CM. Whether neuroinflammation is a cause or consequence of neurodegenerative diseases remains unknown, though activated microglia is known to exacerbate neuronal death^42^. Similarly, we found that LPS CM aggravated the rotenone or MPP+-induced neuronal cell death. We also observe the neuroprotection from the OI CM (Fig. 4E,F,H,I). Interestingly, others have recently reported that itaconate attenuates microglia activation, motor deficits, and dopamine neuronal damage in the MPTP-Induced PD mice model^43^. They also found that direct itaconate treatment attenuated the MPP+-induced apoptosis in SH-SY5Y cells. We did not see any direct protection from OI which is a cell-permeable derivative of itaconate against either rotenone or MPP+ in N2a cells (Fig. 4G). It remains unclear whether OI used in our study fully mimics the effects of endogenous itaconate; though it is a much more potent activator of Nrf2. Further research on itaconate and different derivatives is required to comprehend the function and mechanism of action of these metabolites. Notwithstanding its limitation, itaconate derivatives still provide a viable avenue for studying the role of itaconate in PD models. This is similar to dimethyl fumarate, a derivative of the TCA cycle metabolite fumarate, that contributes to the understanding of the biochemical mechanisms of endogenous fumarate, as well as having potential immunomodulatory effects and being clinically approved for the treatment of inflammatory diseases^44^. LPS stimulation in macrophages has been shown to break the TCA cycle and induce the production of itaconate^45,46^. It is possible that the exogenous OI restored the microglial function by regulating the TCA cycle. Our results of OI’s ability to curb neuronal degeneration via microglia cells call for future research to pursue itaconate and its derivatives as a therapeutic strategy against PD.

In conclusion, our present study provides compelling evidence that OI exerts an anti-inflammatory effect on microglia, conferring protection against toxin-induced neuronal cell death in vitro. The mechanism is associated with the P62/Nrf2/HO-1/NF-κB axis pathway in microglia. Our findings support the concept that itaconate or its analogs hold considerable potential as candidate therapeutics for PD. Further studies are warranted in vivo PD models to evaluate the preclinical safety and efficacy of itaconate-like agents in their development as novel drug candidates for PD.

## Materials and methods

### Cell culture, treatments, and conditioned medium preparation

BV2 and N2a cells were purchased from ATCC and maintained in Dulbecco's Modified Eagle Medium/Nutrient Mixture F-12 (DMEM/F-12) (Thermo Fisher) with 10% fetal bovine serum (Thermo Fisher) at 37 °C with an atmosphere of 5% CO_2_. OI (#6662, Tocris) was dissolved in DMSO (Sigma) at a concentration of 50 mM for stock solution. LPS (Sigma) was dissolved in distilled water at 100 μg/ml for stock solutions. Rotenone (#R8875, Sigma) was dissolved in DMSO at a concentration of 50 mM for stock solution. MPP+ (#D048, Sigma) was in distilled water at a concentration of 1 mM for stock solution and avoid repeated freeze and thaw cycle. Prepare a fresh working solution every time before the treatment.

### Conditioned medium collection

BV2 cells were plated into 60 mm dishes at a density of 8 × 10^5^ cells per dish and a total volume of 4 ml per dish. The next day, 100 ng/ml LPS with or without OI at different final concentrations (5, 25, 50 μM) or DMSO control was added to the dishes. The final concentration of DMSO in the OI working solution in the cells was 0.1%. DMSO of 0.1% was used as a vehicle control in all cell culture assays. Then the cells were further cultured for 3 h and 24 h to collect CM. The CM was centrifuged to obtain cell-free supernatant and then stored at 4 oC. The conditioned medium was used within 24 h after collection.

### N2a cell culture in the presence of CM

N2a cells were seeded into vessels of different sizes. The next day, the medium was removed, and collected CM from various treatment groups with or without 1 μM Rotenone or 200 μM MPP^+^ was added to cells based on the volume recommeded for the specific plates for 24 h before performing downstream assays.

### Measurement for cytokines and nitrite levels

BV2 cells were plated at a density of 3 × 10^5^ per well into 6-well plates. The next day cells were treated with LPS with or without OI for 3 h or 24 h; the supernatant was collected and centrifuged to eliminate dead cells. The production of NO in the supernatant was measured using Griess reagent (Thermo Fisher) according to the manufacturer's instructions. The concentration of inflammatory mediators, including IL-6 and TNF-α were determined by ELISA kits (R&D Systems) according to the manufacturer’s instructions.

### SDS/PAGE Western Immunoblot

BV2 cells were seeded into 6-well plates at a density of 3 × 10^5^ per well. Proteins were collected with M-PER™ Mammalian Protein Extraction Reagent (Thermo Fisher) after 3 h or 24 h incubation with indicated treatment. N2a cells were seeded into 6-well plates at a density of 4 × 10^5^ per well. The next day, N2a cells were exposed to indicated CM for 24 h as mentioned above before protein collection. Western blot was performed as described (4). Primary antibodies used were as follows: COX-2 (1:1000; Cell signaling), iNOS (1:1000; Cell Signaling), α/β tubulin (1:2000; Cell Signaling), Nrf2 (1:500; Cell Signaling), HO-1 (1:4000; Cell Signaling), phosphorylated-P62 (1:2000; Cell Signaling), total P-62 (1:2000; Cell Signaling), cleaved caspase-3 (1:1000; Cell Signaling), caspase-3 (1:1000; Cell Signaling),or β-actin (1:2000; Cell Signaling). A horseradish peroxidase-conjugated secondary antibody (1:10,000; Cell Signaling) and a Supersignal West Femto Maximum Sensitivity Substrate kit (Thermo Fisher) were used for detection. The density of the bands was analyzed using ImageStudioLite software.

### Fluorescence microscopy

BV2 cells were seeded on 8-well chamber slides (#80841, ibidi) at a density of 2 × 10^4^ per well. The next day, cells were treated as indicated. Then immunofluorescence assays were all performed according to our standard protocol as described before^47^. Primary antibodies used were Nrf-2 (1:250; Cell Signaling), Nf-kB (1:250; Cell Signaling), phosphorylated-P62 (1:500; Cell Signaling), and DAPI (1 μg/ml, Thermo Fisher). Images were collected with a Nikon A1+/A1R+ confocal microscope.

### Cell viability assays

N2a cells were seeded in 96-well plates at a density of 2.0 × 10^4^ cells per well and a total volume of 200 μl. The next day, the medium was removed, and 200 μl CM with or without toxins was added to each well for a further 24 h incubation. Cell viability was examined by an MTS assay kit (Thermo Fisher) according to the manufacturer’s instructions.

### Statistics

Values were expressed as mean ± standard error of the mean (SEM). Differences between groups were examined for statistical significance using one-way ANOVA using GraphPad Prism 8 software. A *p*-value less than 0.05 denoted the presence of a statistically significant difference, S2.

### Supplementary Information

Supplementary Figures.

## Data Availability

The datasets generated and/or analyzed during the current study are not publicly available but are available from the corresponding author upon reasonable request.
