# Supplementary material for: Microglia-dependent neuroprotective effects of 4-octyl itaconate against rotenone-and MPP+-induced neurotoxicity in Parkinson’s disease
Source: Sci Rep. 2023 Sep 20;13:15539. doi: 10.1038/s41598-023-42813-8 (PMC10511514; doi:10.1038/s41598-023-42813-8)
Supplement: Supplementary file 1 — Supplementary Figures. [file 41598_2023_42813_MOESM1_ESM.pdf]

Microglia-dependent neuroprotective effects of 4-octyl itaconate against rotenone-and MPP + -induced neurotoxicity in Parkinson's disease

* Ning Xia[1,2], Victoria Madore[1], Ali Albalakhi[1], Sonia Lin[1], Taylor Stimpson[1], Yuehang Xu[1], Michael A. Schwarzschild[1,2], Rachit Bakshi[1,2]

[1] Molecular Neurobiology Laboratory, Massachusetts General Hospital, Boston, MA 02129, USA

[2] Harvard Medical School, Boston, MA 02115, USA

Supplemental data

Figure 1.

A

B

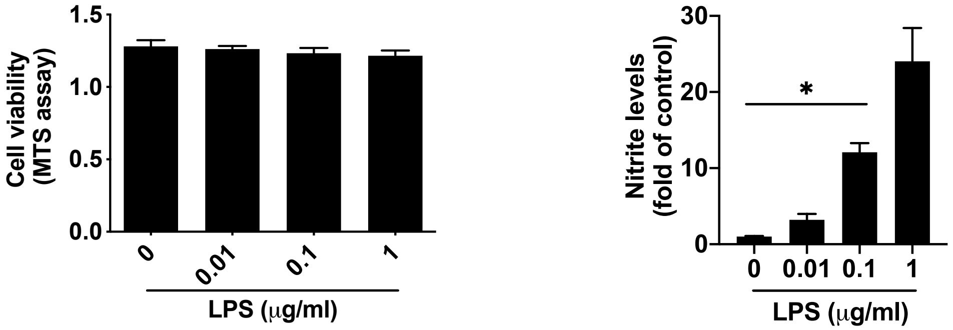

Figure 1. Dose effect of LPS on BV2 Cell viability and nitrite production. (A) Cell viability after LPS exposure. Cell viability was tested by MTS assay. BV2 cells were treated as indicated for 24 h. (B) Nitrite levels were tested by Griess assay after treatment as indicated for 24 h. (mean ± SEM, one-way ANOVA).

Figure 2.

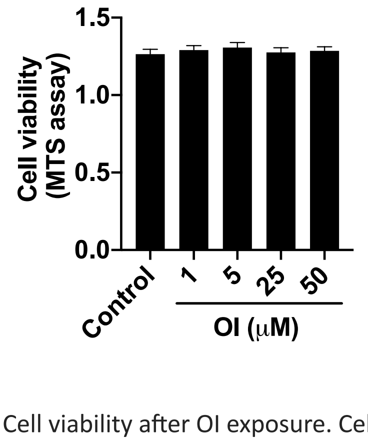

Figure 2. Cell viability after OI exposure. Cell viability was tested by MTS assay. BV2 cells were treated as indicated for 24 h (mean ± SEM, one-way ANOVA)
